# Cell number regulator genes in *Prunus* provide candidate genes for the control of fruit size in sweet and sour cherry

**DOI:** 10.1007/s11032-013-9872-6

**Published:** 2013-04-30

**Authors:** P. De Franceschi, T. Stegmeir, A. Cabrera, E. van der Knaap, U. R. Rosyara, A. M. Sebolt, L. Dondini, E. Dirlewanger, J. Quero-Garcia, J. A. Campoy, A. F. Iezzoni

**Affiliations:** 1 Dipartimento di Scienze Agrarie, Università degli Studi di Bologna, Bologna, Italy; 2 Michigan State University, East Lansing, MI USA; 3 Ohio Agricultural Research and Development Center, The Ohio State University, Wooster, OH USA; 4 INRA, UMR 1332 de Biologie du Fruit et Pathologie, 33140 Villenave d’Ornon, France; 5 University of Bordeaux, UMR 1332 de Biologie du Fruit et Pathologie, 33140 Villenave d’Ornon, France

**Keywords:** Cell number regulator, Fruit size, Marker-assisted selection, Cherry, Domestication, *Prunus*

## Abstract

Striking increases in fruit size distinguish cultivated descendants from small-fruited wild progenitors for fleshy fruited species such as *Solanum lycopersicum* (tomato) and *Prunus* spp. (peach, cherry, plum, and apricot). The first fruit weight gene identified as a result of domestication and selection was the tomato *FW2.2* gene. Members of the *FW2.2* gene family in corn (*Zea mays*) have been named *CNR* (Cell Number Regulator) and two of them exert their effect on organ size by modulating cell number. Due to the critical roles of *FW2.2/CNR* genes in regulating cell number and organ size, this family provides an excellent source of candidates for fruit size genes in other domesticated species, such as those found in the *Prunus* genus. A total of 23 *FW2.2/CNR* family members were identified in the peach genome, spanning the eight *Prunus* chromosomes. Two of these *CNRs* were located within confidence intervals of major quantitative trait loci (QTL) previously discovered on linkage groups 2 and 6 in sweet cherry (*Prunus avium*), named *PavCNR12* and *PavCNR20*, respectively. An analysis of haplotype, sequence, segregation and association with fruit size strongly supports a role of *PavCNR12* in the sweet cherry linkage group 2 fruit size QTL, and this QTL is also likely present in sour cherry (*P. cerasus*). The finding that the increase in fleshy fruit size in both tomato and cherry associated with domestication may be due to changes in members of a common ancestral gene family supports the notion that similar phenotypic changes exhibited by independently domesticated taxa may have a common genetic basis.

**Electronic supplementary material:**

The online version of this article (doi:10.1007/s11032-013-9872-6) contains supplementary material, which is available to authorized users.

## Introduction

Cultivated fruit and vegetable crops often bear little phenotypic resemblance to their wild ancestors (Paran and van der Knaap 2007). The change from a hunter-gatherer to an agricultural lifestyle, starting approximately 10,000–13,000 years ago, led to the domestication of plants from wild progenitors, leading to plants better adapted to cultivation and human use. The resulting selection of alleles from wild progenitors, many of which may have arisen as spontaneous mutations, led to dramatic changes in plant traits associated with the domestication syndrome (Hammer 1984), including increases in the size of edible organs such as fleshy fruit.

Domestication-associated increases in fleshy fruit size occurred in diverse plant families such as the Cucurbitaceae (Nuñez-Palenius et al. 2008; Esteras et al. 2011; Paris et al. 2012), Solanaceae (Tanksley 2004; Paran and van der Knaap 2007; Wang et al. 2008; Meyer et al. 2012) and Rosaceae (Miller and Gross 2011). However, the understanding of the genetic changes that resulted in this fruit size increase between domesticates and their small-fruited wild relatives is most advanced in tomato (*Solanum lycopersicum* L.) (Grandillo et al. 1999; Brewer et al. 2007; Paran and van der Knaap 2007; Causse et al. 2007; Gonzalo and van der Knaap 2008). One of the major tomato fruit size quantitative trait loci (QTL) explained approximately 30 % of the fruit weight variation in interspecific populations (Alpert and Tanksley 1996). The underlying gene, *FW2.2*, was identified by map-based cloning and shown to be expressed in the early stages of fruit development and to modulate cell proliferation (Frary et al. 2000; Cong et al. 2002). *FW2.2* copy number and expression levels were negatively correlated with cell division activity in the early stages of fruit development; therefore, *FW2.2* was proposed to act as a negative regulator of cell number (Liu et al. 2003). Members of the *FW2.2* gene family have been identified in other plants such as avocado fruit (*Persea americana* Mill.) (Dahan et al. 2010) and soybean root nodules [*Glycine max* (L.) Merr.] (Libault et al. 2010), where they are hypothesized to control cell number. In maize (*Zea mays* L.), a genome-wide search for *FW2.2* family members led to the identification of a family of 13 genes, named cell number regulators (*CNR*; Guo et al. 2010). The over-expression of *ZmCNR1* resulted in a reduction of overall plant stature, by acting as a negative cell number regulator in multiple tissues, while *ZmCNR2* also affected organ and plant size (Guo et al. 2010).

*FW2.2* and *CNR* genes share a cysteine-rich domain named PLAC8, first characterized in mammalian placenta (Galaviz-Hernandez et al. 2003), whose function is unknown. In addition to their involvement in the regulation of cell proliferation, members of the PLAC8 family have been characterized as membrane-bound proteins capable of interacting with metal cations. Among these, PCR (Plant Cadmium Resistance) genes are involved in extrusion of cadmium and zinc ions through the plasma membrane, contributing to heavy metal detoxification (Song et al. 2004, 2010); and MCA (Mid1-Complementing Activity) genes were identified for their ability to restore calcium uptake in yeast cells lacking the Mid1/Cch1 channel (Yamanaka et al. 2010).

The *Prunus* genus in the Rosaceae family includes many fleshy-fruited species such as peach [*Prunus persica* (L.) Batsch], diploid sweet cherry (*Prunus avium* L.), tetraploid sour cherry (*P. cerasus* L.), plum (*P*. *domestica* L. and *P. salicina* Lindl.) and apricot (*P. armeniaca* L.) that are cultivated in temperate regions throughout the world. Cultivars that consistently produce large fruits are critical for grower profitability. For example, for the fresh-market sweet cherry, fruit size is the main criterion by which the fruit is graded for sale (Whiting et al. 2006). Therefore, obtaining new large-fruited cultivars is a major breeding goal. However, improvement of *Prunus* fruit tree crops has lagged behind annual crops, in part due to the long juvenile phase that can last up to 5 years and significantly hampers the expeditious phenotypic evaluations for fruit quality traits. Therefore, knowledge of markers and genes associated with fruit size in *Prunus* species has the potential to significantly increase the efficiency of breeding large-fruited cultivars, as it would allow the early elimination of seedlings that have the potential of bearing fruit that is smaller than the target size threshold. This knowledge would also greatly facilitate the use of small-fruited wild germplasm, as it would reduce the number of generations needed to obtain the commercial fruit size needed for a new cultivar.

In sweet cherry, the wild, landrace and modern varieties typically exhibit fruit weights of 2 g, 6 g, and up to 14 g, respectively. Although fruit weight in *Prunus* behaves as a quantitative trait like that of tomato fruit weight, a high portion of the phenotypic variation is explained by a few major QTL (Zhang et al. 2010). In a cross between a wild mazzard, New York 54, and a landrace sweet cherry, Emperor Francis, QTL were identified on linkage groups 2 (G2) and 6 (G6), with the G2 QTL postulated to affect fruit size by controlling mesocarp cell number (Zhang et al. 2010). In tetraploid sour cherry, whose ancestral sub-genomes are derived from both the diploid sweet cherry and the wild tetraploid ground cherry (*P. fruticosa* Pall.), a G2 QTL was identified in a similar linkage group position (Wang et al. 2000).

The *CNR* gene family provides an excellent source of candidate genes for investigating the genetic control of fruit size in *Prunus*. Its critical role of controlling fruit size by increasing cell number and organ size is demonstrated by *FW2.2* in tomato (Frary et al. 2000), and *ZmCNR1* and *ZmCNR2* in maize (Guo et al. 2010). Using the peach genome v.1.0 sequence released by the International Peach Genome Initiative (GDR database: http://www.rosaceae.org/species/prunus_persica/genome_v1.0), the identification of the *CNR* family in *Prunus* is possible. Because of the high level of synteny between peach and the other *Prunus* species (Dirlewanger et al. 2004; Cabrera et al. 2009; Jung et al. 2009; Illa et al. 2011; Klagges et al. 2013), peach can serve as a model genome for the genus. In the present study, we identified the peach *CNR* gene family and investigated the possibility that two members are candidates for the control of two fruit size QTL in cherry.

## Materials and methods

### Identification of *CNR* gene family members in the peach genome

The protein sequences of tomato FW2.2 (Frary et al. 2000) and maize CNRs (Guo et al. 2010) were retrieved from NCBI (http://www.ncbi.nlm.nih.gov/). To identify the *CNR* family members in the peach genome sequence v1.0 (International Peach Genome Initiative; http://www.rosaceae.org/peach/genome), the algorithm BLASTP was used. The genes were named *PpCNR* (*P. persica* *Cell Number Regulator*) followed by a number, based on their order on the peach genome scaffolds. Their predicted protein sequences were retrieved and aligned to known FW2.2/CNR proteins using the set of animal, fungi and plant sequences analyzed by Guo et al. (2010). Additional tomato *FW2.2/CNR* genes were identified from SGN (http://solgenomics.net/), resulting in 19 tomato members presumably representing the entire family. Also included were other recently published *CNR*-like genes, viz. avocado *Pafw2.2*-*like* (Dahan et al. 2010), soybean *GmFWL1* (Libault et al. 2010) and tobacco *NtMCA1* and *NtMCA2* (Kurusu et al. 2012). Sequence alignment and phylogenetic analyses were conducted using MEGA version 5 (Tamura et al. 2011). The protein sequences were aligned by ClustalW using the BLOSUM protein weight matrix and gap opening and extension penalties of 10 and 0.1, respectively. A neighbor-joining tree was then built using the Poisson substitution model and uniform rates, and statistical support was obtained by bootstrap analysis with 1,000 replicates.

### Plant materials

The first of two sweet cherry segregating F1 populations used in this study, N × E, consisted of 557 individuals derived from reciprocal crosses between the large-fruited landrace cultivar Emperor Francis (E) and the small-fruited, wild mazzard genotype New York 54 (N), and is maintained at the Michigan State University’s Clarksville Research Center in Clarksville, MI, USA. The second F1 population, R × L, consisted of 133 individuals obtained from the cross between the cultivars Regina (R) and Lapins (L), and grown at the Institute National de la Recherche Agronomique in Bordeaux, France. Subsets of both of these populations have been previously used for genetic linkage map construction and the mapping of fruit weight QTL (N × E: Olmstead et al. 2008; Zhang et al. 2010; R × L: Dirlewanger et al. 2004; 2009). In addition, a set of 17 sweet cherry cultivars, previously determined to reflect the range of diversity in sweet cherry germplasm (Cabrera et al. 2012), was used to assess allelic variation of the *CNR* candidate genes identified. Four of the 17 selections were the parents of the two sweet cherry F1 populations used in this study.

In sour cherry, five bi-parental F1 populations were evaluated. The largest population, M172 × 25-02-29, consisted of 79 individuals, followed by Újfehértói Fürtös × Surefire (*n* = 72); 25-14-20 × 25-02-29 (*n* = 57); Montmorency × 25-02-29 (*n* = 36); and Rheinische Schattenmorelle (RS) × Englaise Timpurii (ET) (*n* = 22), totaling 274 individuals including parents. All sour cherry individuals are maintained at the Michigan State University’s Clarksville Research Center in Clarksville, MI, USA.

### Trait measurements

Phenotypic data for the sweet cherry N × E and R × L populations were collected for 3 years (2009–2011 and 2008–2010, respectively). For the N × E population, in 2009, phenotyping was performed on all the fruiting plants (*n* = 436), while in 2010 and 2011 phenotyping was conducted on those N × E progeny individuals that carried a recombination breakpoint in the fruit weight QTL interval on G2 to enable more precise mapping of the fruit weight QTL. Fruit weight of the N × E progeny individuals was measured by weighing five individual fruit that were collected twice and the mean weight was calculated for both collections. For the N × E population, mesocarp cell number data that was previously collected in 2006 and 2008 and used to identify the cell number QTL on cherry G2 overlapping with the fruit size QTL (Zhang et al. 2010) was also used. To calculate flesh weight in the N × E progeny, fruit weight and pit weight were recorded for each fruit in 2011. Flesh weight was calculated by subtracting pit weight from total fruit weight for each fruit. For the R × L progeny, the mean weight of 50 fruit was measured for all the individuals that could be harvested (*n* = 104, *n* = 116 and *n* = 114 in 2008, 2009 and 2010, respectively). For sour cherry, fruit and pit weights were measured for each of five individual fruit that were collected twice and the mean weight was calculated. Mean flesh weight was calculated by subtracting the mean pit weight from the mean fruit weight for these same five fruit.

### Sequencing of candidate fruit weight *CNR*s in cherry

Taking advantage of the synteny between the peach and cherry genomes, and the presence of conserved markers on the peach and cherry genetic maps (G2: CPSCT038, BPPCT034; G6: PR86), the genomic regions of peach corresponding to the two sweet cherry fruit size QTL were identified (Zhang et al. 2010). A peach *CNR* gene was found within each of these G2 and G6 regions, *PpCNR12* and *PpCNR20,* respectively. Whole-genome shotgun sequences of four sweet cherry (New York 54, Emperor Francis, Attika and Napoleon, at 2.5×, 3.9×, 3.8× and 2.1 × coverage, respectively), and two sour cherry (Rheinische Schattenmorelle and 23-23-13, at 2.3× and 0.7 × coverage, respectively) genotypes were obtained to identify the best cherry ortholog sequences for *PpCNR12* and *PpCNR20*. Contig fragments (300 bp on average) were built de novo with Velvet (http://genome.cshlp.org/content/18/5/821.short) with optimized parameters using a subset of 76-bp paired-end reads that correspond to the G2 and G6 fruit weight QTL regions from peach. Consensus cherry contigs corresponding to the genomic regions of *PpCNR12* and *PpCNR20* were obtained and used for primer development. Six primer pairs (CNR12-C1 to CNR12-C6 and CNR20-C1 to CNR20-C6, Supplementary Table S1) designed to amplify fragments of approximately 700–1,100 bp, and tailed with the M13F and M13R sequences to facilitate high-throughput sequencing, were used to sequence each gene region.

PCR reactions were conducted using the following parameters: 1 × PCR buffer, 0.2 mM each dNTP, 1.5 mM MgCl_2_, 0.5 μM each primer, 2 ng/μL genomic DNA and 0.02 U/μL *Taq* DNA polymerase (Invitrogen, Carlsbad, CA, USA). Cycling conditions were as follows: initial denaturation for 3 s at 95 °C; 30 cycles of annealing for 45 s at 60 °C, extension for 90 s at 72 °C and denaturation for 30 s at 95 °C; and a final extension for 10 s at 72 °C. PCR products were separated using electrophoresis and visualized on a 1.2 % agarose gel, and amplicon concentration was estimated by comparison with the closest band of a 100-bp and 1-kb DNA ladder (New England Biolabs, Ipswich, MA, USA). Purification of PCR products was carried out using ExoSAP-IT (Affymetrix, Santa Clara, CA, USA) according to the manufacturer’s instructions and the PCR amplicons were sequenced using M13 forward and reverse sequencing primers at the Michigan State University Research Technology Support Facility. Sequencher 5.0 (Gene Codes Corporation, Ann Arbor, MI, USA) was used to align the reads and call double peaks corresponding to heterozygous SNP positions. The two cherry genes were named *PavCNR* (for *P. avium CNR*) *12* and *20*.

### Analysis of *PavCNR12* and *PavCNR20* allelic variation in sweet cherry

The sequences for the *PavCNR12* alleles and upstream regions were deduced by sequencing the parents, the representatives of the homozygous individuals from the N × E and R × L progenies, and the 17 diverse sweet cherry cultivars to determine the sequence of each haplotype. Sequences were aligned to the peach ortholog and the coding sequence was deduced accordingly. The sequences were also analyzed using TSSP (Solovyev and Shahmuradov 2003) to predict the transcript start site. Subsequently, the *PavCNR12* alleles were distinguished by the sequences of fragment CNR12-C2, which contained six polymorphic sites differentiating the three alleles. Sequencing of the C2 fragment was carried out for all of the progeny individuals showing recombination or ambiguities in the region between CPSCT038 and BPPCT034 (Cabrera 2011), plus a number of non-recombinant individuals representing all the G2 QTL genotypic classes.

Sequencing of *PavCNR20* alleles was performed with DNA from New York 54, Emperor Francis, Ambrunes and Cristobalina using primers tailed with the M13F and M13R sequences (Supplementary Table S1). However, attempts to obtain the full-length sequence for *PavCNR20* failed due to the presence of insertion/deletion polymorphisms hampering the read of chromatograms obtained from amplification of heterozygous genotypes. Consequently, the sequence information obtained covered only a non-contiguous portion of the gene. Interestingly, one of the primer pairs (CNR20–C1, Supplementary Table S1) only amplified a fragment from New York 54, highlighting the presence of a unique allele in this genotype; this primer pair was then used to assay the presence of the same allele in the remaining set of 13 sweet cherry cultivars.

### Fine mapping of the *PavCNR12* region in sweet cherry

A total of nine simple sequence repeat (SSR) primer pairs were developed in silico from the peach genomic region syntenic to the sweet cherry G2 fruit weight QTL region previously described (Zhang et al. 2010) (G2SSR1576, G2SSR1580, G2SSR1610, G2SSR1672, G2SSR1678, G2SSR1675, G2SSR1818, G2SSR1823, G2SSR1864; Supplementary Table S2). The SSRs were identified from regions close to predicted genes using SSRIT (Temnykh et al. 2001) and WebSat (Martins et al. 2009). Flanking primers were developed using Primer3 v0.4.0 (Rozen and Skaletsky 2000; http://frodo.wi.mit.edu/primer3) and a M13 tail (CACGACGTTGTAAAACGAC) was added to the 5′ end of all of the forward primers to facilitate labeling of products during the PCR reaction. In addition, a primer pair for one SNP marker (G2SNP1623, Supplementary Table S2) identified after sequencing a peach intergenic region was designed for genotyping using the allele-specific primer extension (ASPE) method using the Luminex technology (Luminex, Corp., Austin, TX, USA). A polymerase-mediated primer extension identified the base at a specific SNP on a previously amplified product (Supplementary Table S2). Uniquely colored microspheres were attached to specific products and the fluorescence of a reporter molecule (streptavidin) was quantified by a laser in a Luminex 200 analyzer (Lee et al. 2004).

SSRs were run on a 6.5 % LI-COR KB^Plus^ gel (LI-COR, Lincoln, NE, USA). The reaction mixture for the SSR amplification contained 1 × PCR buffer, 2 mM MgCl_2_, 100 μM of each dNTP, 0.02 μM of each primer, 1 μM LI-COR primer (IRDye700 or IRDye800) and 0.3 U/μL *Taq* DNA polymerase. Conditions for PCR amplification were as follows: initial denaturation at 94 °C for 1 min; 31 cycles of 92 °C for 40 s, 56 °C for 45 s and 72 °C for 2 min, and a final extension at 72 °C for 4 min. Fragments were detected by excitation of fluorescence added during the PCR reaction with either IRDye700 or IRDye800 primers following the M13-tailed PCR protocol (Schuelke 2000). A total of 549 individuals from the N × E population and 133 individuals from the R × L population were genotyped with these new markers at the Molecular and Cellular Imaging Center in Wooster, OH, USA. Map distances for markers in the G2 QTL region were calculated using JoinMap 3.0 (Van Ooijen and Voorrips 2002).

### Genotyping markers linked to *PcrCNR12* and *PcrCNR20* in sour cherry

Orthologs of *PavCNR12* and *PavCNR20,* named *PcrCNR* (for *P. cerasus* *CNR*) *12* and *20*, were amplified from sour cherry using the same primer sets used for sweet cherry (Supplementary Table S1). However, sequence alignments of amplicons from sour cherry were not possible due to the tetraploid and highly heterozygous nature of the genomic regions. Instead, the peach genome was utilized to identify SSRs in transcripts near the genes *PpCNR12* and *PpCNR20*. Primer3 was used for primer design. PCR fragments for the G2- and G6-associated SSRs (G2SSR1566 and G6SSR2208, respectively; Supplementary Table S2) were amplified, separated on 5 % polyacrylamide gels and visualized with silver staining. When the SSR fragments sizes for sour cherry were equivalent to those for sweet cherry, flanking SNP genotypes previously obtained using the Cherry 6 K Infinium^®^ II array (Peace et al. 2012) were used to determine allele identity.

### Statistical analysis

To test the likelihood of *PavCNR12* as the underlying candidate gene for the G2 QTL, an analysis of variance (ANOVA) implemented in R stat version 2.15.1 (R Development Core Team, 2012) was conducted. ANOVA was performed using the following general linear model, in which both the allele main effects and interactions were tested for significance:

$$ {\varvec{Y}} = {\varvec{X\beta}} + {\varvec{\varepsilon}} $$

where ***Y*** is a vector (*n* × 1) of observed phenotypic values for *n* individuals, ***X*** is a customized design matrix with *n* × *p* fixed constants for all allele main effects and multi-way interactions, and *p* is the number of *x* parameters (both fixed main and interaction effects). The alleles were assumed to be additive. Therefore, for the diploid (sweet cherry) case, 0, 1 and 2 were assigned for absence, single and two dosages of a particular allele, respectively. For the tetraploid (sour cherry) case 0, 1, 2, 3 and 4 were assigned for absence, single, two, three and four dosages of particular alleles, respectively. Both main-effect and higher level interactions were considered. ***β*** is a *n* × 1 unknown fixed effects parameter vector. ***ε*** is a *n* × 1 vector of residuals (random errors). Kruskal–Wallis tests were also conducted, resulting in similar results to the ANOVA. *P* values <0.05 were reported as significant. Data for 3 years from each sweet cherry population were initially analyzed separately; then, to combine data from different years, values within each year were standardized using the following formula:

$$ x_{\text{std}} = \frac{{(x - \bar{x})}}{\sigma } $$

where $\bar{x}$ and *σ* are the mean and standard deviation of the data in that year; the mean of standardized values was then calculated for each genotype, obtaining a single dataset for each trait.

The mean trait comparisons among genotypic classes for *PavCNR12* and *PavCNR20* in sweet cherry were done using the Newman–Keuls test implemented in SAS version 9.0 (SAS Institute, Cary, NC, USA). In the case of tetraploid sour cherry, a two-tailed Student’s *t* test was used to compare the trait means of two classes, e.g. individuals with the putative allele versus individuals without the putative allele for the SSR marker loci closely linked to both *CNR* candidate genes.

The threshold for significance was set at *P* < 0.05.

## Results

### Identification of the peach *CNR* gene family members and candidate genes for cherry fruit size QTL

**Fig. 1:**
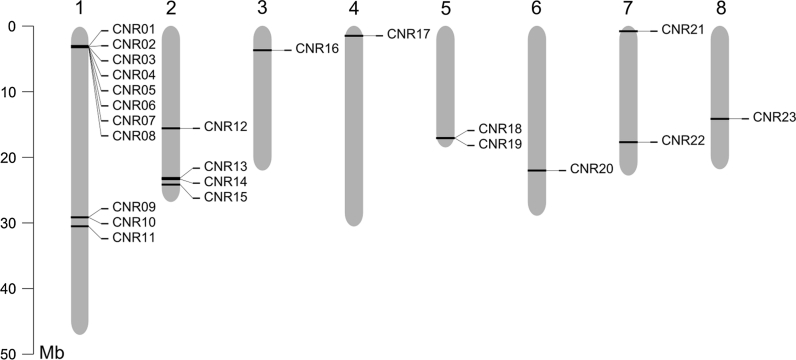
Position of the 23 *CNR* homologs identified in the eight peach genome scaffolds

**Fig. 2:**
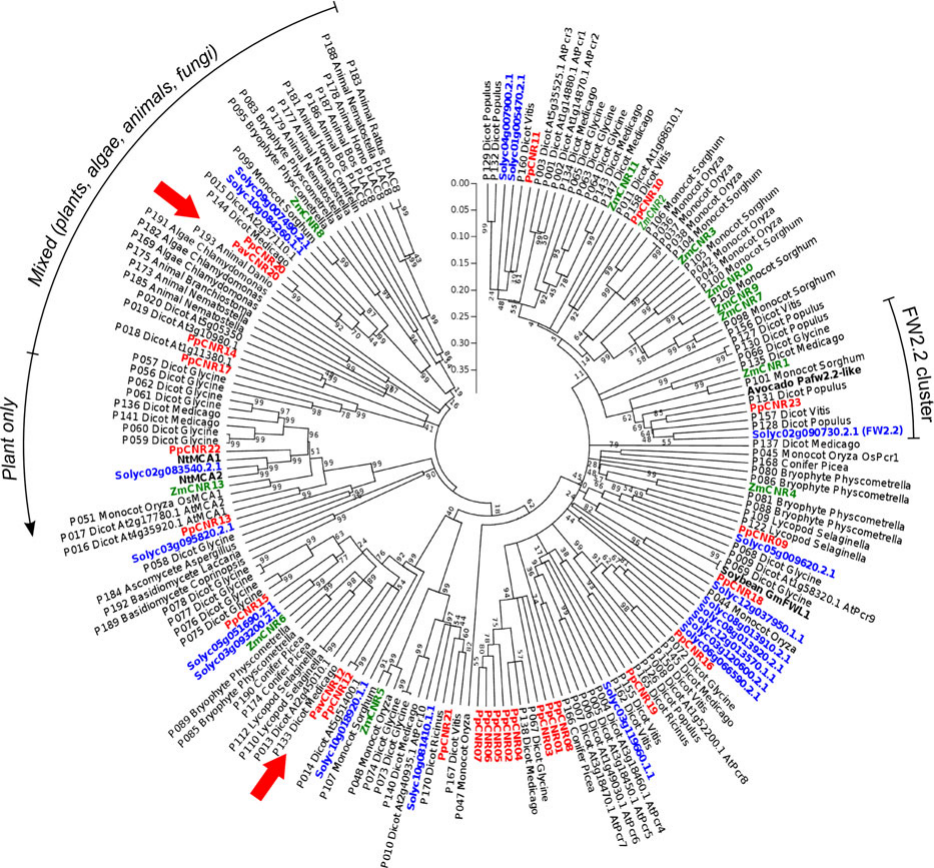
Neighbor-joining tree of *FW2.2/CNR* homologs from diverse taxa (derived from Guo et al. 2010). The tree includes the entire families identified in maize (*green*), tomato (*blue*) and *Prunus* (*red*); the two candidates for the control of fruit size in cherry (*PavCNR12* and *PavCNR20*) are indicated by *red arrows*. (Color figure online)

A total of 23 *CNR* gene family members were identified in the peach genome, with at least one *CNR* gene identified on each of the eight chromosomes (Supplementary Table S3 and Fig. 1). The structure for most of the peach *CNR* genes consisted of two or three introns; however, one gene had four introns (*PpCNR17*), two genes had six introns (*PpCNR13* and *22*), and one gene was intronless (*PpCNR14*) (Supplementary Fig. S1). The deduced protein sequences ranged from 84 to 445 residues, with the majority of the *CNR*s (16 out of 23) consisting of between 100 and 255 amino acids. While each of the eight peach chromosomes contained *CNR* family members, chromosome 1 had the most, 11 *CNR*s. Eight of the *CNR*s on chromosome 1 (*PpCNR01* to *08*) formed a dense cluster between 3.056 and 3.139 kb (Supplementary Table S3 and Fig. 1). The phylogenetic analysis carried out on these deduced protein sequences (Fig. 2) indicated homology between these eight genes, suggesting that they likely originated from a series of recent tandem duplication events.

The deduced peptide sequences of the 23 PpCNRs had amino acid identities with the closest FW2.2/maize CNRs ranging from 21.0 to 65.6 %. The composition of the PpCNRs tended to be rich in cysteine and proline, with an average of 7.40 % for both amino acids. Three proteins (PpCNR9, 15 and 18) contained the CLXXXXCPC conserved motif found in the cluster of cell-number regulating proteins containing FW2.2 and ZmCNR1. Three other proteins (PpCNR10, 11 and 21) contained the motif CCXXXXCPC, reported for ZmCNR2 and some cadmium resistance-related proteins. Finally, in three other peptides (PpCNR12, 17 and 23) this motif is partially conserved as CXXXXXCPC. The peach homolog most similar to tomato FW2.2 and maize ZmCRN1 was PpCNR23 (Fig. 2).

**Fig. 3:**
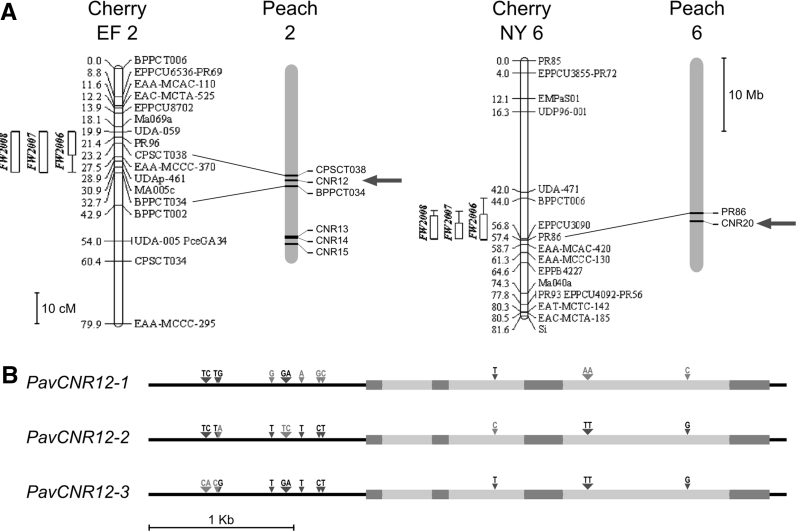
**a** Position of fruit weight (FW) QTL in sweet cherry G2 and G6 (from Zhang et al. 2010) and identification of the corresponding regions in peach scaffolds; two *CNR* genes whose position is compatible with the QTL region are indicated by *arrows*. **b** Polymorphisms differentiating the three *PavCNR12* alleles. The coding sequence is represented in *thick dark grey bars*, and introns in *light gray*

To investigate whether peach *CNR* genes were located within the sweet cherry fruit size QTL regions, the sequence between the two SSR markers that flank the G2 fruit weight QTL, CPSCT038 (at 15.057 Mb) and BPPCT034 (at 16.491 Mb), was examined. *PpCNR12* (identified in the peach genome as transcript ppa026136 m) was identified on scaffold 2 at approximately 15.650 Mb (Supplementary Table S3) and therefore qualified as a likely candidate gene for the G2 fruit size QTL (Fig. 3a). The G6 fruit weight QTL was near marker PR86 (Zhang et al. 2010), which is located approximately 1 Mb from *PpCNR20* (ppa008853 m) (Supplementary Table S3, Fig. 3a). Therefore, *PpCNR20* is a likely candidate gene for the G6 fruit size QTL.

### Identification of *PavCNR12* allelic variants

The consensus sequence of *PavCNR12* consisted of 4,375 kb, including 1,491 bp upstream of the start codon and 117 bp downstream of the stop codon. The coding region, which is 768 bp long, was interrupted by three introns of 341, 517 and 1,141 bp. The deduced protein sequence of PavCNR12 has 255 residues with an amino acid identity of 97.6 % with its peach ortholog PpCNR12 and 66.8 % with maize ZmCNR6. The percentages of cysteine and proline residues are 6.67 % and 7.45 %, respectively, and the protein is predicted to harbor the CXXXXXCPC motif.

To determine the allelic variation for *PavCNR12*, the gene and upstream region were sequenced from 17 sweet cherry cultivars chosen to represent a diverse array of sweet cherry germplasm (Cabrera et al. 2012, Supplementary Table S4). The sequenced *PavCNR12* fragments revealed no polymorphisms within the coding regions and 14 nucleotide polymorphisms in the non-coding regions that collectively distinguished three unique *PavCNR12* sweet cherry alleles (Fig. 3b, Supplementary Figure S2). Of these, 10 polymorphisms resided in the 5′ region upstream of the start codon, one in the second intron and three in the third intron (Fig. 3b). Seven out of 17 sequenced cultivars were homozygous for the most prevalent allele, named *PavCNR12*-*1* (Supplementary Table S4). A second allele, *PavCNR12*-*2,* was identified in both Regina and Lapins and its haplotype was confirmed by sequencing R × L progeny individuals homozygous for this second allele (Supplementary Table S4). The same two alleles identified in Regina and Lapins were also present in Emperor Francis. Finally, a third allele (*PavCNR12*-*3*) was identified in New York 54. The sequencing of the *PavCNR12* haplotypes in the 17 founder lines allowed us to determine the allelic composition in 16 sweet cherry cultivars. The only unique haplotype was found for Cristobalina, which showed a SNP in the third intron of the otherwise *PavCNR12*-*3* haplotype (Supplementary Figure S2). Therefore, we considered Cristobalina to carry the *PavCNR12* 1/3 alleles (Supplementary Table S4).

As most of the polymorphic sites were located in the region between 1,100 and 300 bp upstream of the start codon, the sequences of the three alleles were analyzed by TSSP to predict promoter and enhancer elements. For *PavCNR12*-*1* the putative transcription start site (TSS) was placed at position 1,077 (−415 from the start codon), with a TATA box at 1,044 (−448). For *PavCNR12*-*2* and *PavCNR12*-*3*, the TSS was placed closer to the start codon, at position 1,174 (−318), with the TATA box at 1,151 (−341). The sequences of *PavCNR12*-*1*, *PavCNR12*-*2* and *PavCNR12*-*3* were submitted to Genbank (accession numbers KC139086, KC139087 and KC139088, respectively).

### Identification of *PavCNR20* allelic variants

In sweet cherry, the fragments from Emperor Francis, Ambrunes and Cristobalina resulting in readable sequences were from CNR20-C4 through C6. This yielded a contig of approximately 1.8 kb, corresponding to the 3′ portion of the gene and encoding 141 C-terminal amino acids. No polymorphisms were found in this region between the three cultivars and the sequence was submitted to Genbank (accession number KC154001). C1 primer pairs did not amplify a product, and C2 and C3 resulted in double peaks indicative of several indels close to the primer sites in these three cultivars. From New York 54, only fragment CNR20-C1 could be amplified and sequenced. All other fragments from the wild mazzard resulted in unreadable chromatograms. In both New York 54 and the cultivars, the *CNR20* alleles either were divergent or primer pairs amplified two closely related paralogs rather than a single gene. Nevertheless, it appeared that the two New York 54 alleles for *CNR20* are more diverse than those in the cultivars.

Since the G6 sweet cherry fruit weight QTL was only segregating in the New York 54 parent (Zhang et al. 2010), we sought to determine whether any of the sweet cherry founder lines carried the small fruit allele from the wild mazzard. The primer pair CNR20-C1 was used to test the presence of this allele and showed that none of the 16 sweet cherry cultivars resulted in amplification of this fragment, suggesting that the small fruit allele is unique to New York 54.

### Association of *PavCNR12* allelic variants with phenotypic variation

The *PavCNR12* genotypes of New York (*1/3*) and Emperor Francis (*1/2*), were consistent with those predicted from the previous QTL analysis where both parents were shown to share one common G2 QTL haplotype while each possessing a second unique QTL haplotype (Zhang et al. 2010). The heterozygous *PavCNR12* genotypes found for Lapins (*1/2*) and Regina (*1/2*), were also consistent with the previous finding that both parents were heterozygous for the G2 fruit size QTL (Dirlewanger et al. 2009).

To determine whether the three *PavCNR12* alleles were associated with fruit size variation in sweet cherry, the segregation was first analyzed in the two sweet cherry F1 populations. In N × E, the four genotypic classes for *PavCNR12* were (*1/1*):(*1/2*):(*1/3*):(*2/3*) and segregated in the ratio 125:136:115:170, while in R × L the ratio between the three classes (*1/1*):(*1/2*):(*2/2*) was 23:61:37. The genotypic frequencies did not differ significantly (*P* > 0.05; *χ* ^2^ test) from the expected ratios of 1:1:1:1 and 1:2:1, respectively. In N × E, mean fruit weights were significantly different (*P* < 0.05) depending on the *PavCNR12* genotype present in progeny individuals (Table 1). Progeny individuals with the genotypes *PavCNR12*-*1/1* and -*2/3* consistently showed the highest and the lowest fruit weight means, respectively. However the fruit weight difference between *PavCNR12*-*1/1* progeny individuals and the second largest class (*PavCNR12*-*1/2* progeny individuals) was significant only in one out of 3 years (2011), while *PavCNR12*-*2/3* progeny individuals had significantly smaller mean fruit weights than any other genotypic group in 2009 and 2010. Of the remaining genotypes, progeny with *PavCNR12*-*1/2* had a slightly higher mean fruit weight than *PavCNR12*-*1/3* progeny individuals, and in 1 year (2009) the difference between the two groups was significant.

**Table 1:** Phenotypic means for *PavCNR12* genotypic classes of progeny individuals from the populations New York 54 × Emperor Francis (N × E) and Regina × Lapins (R × L)

Population	Trait means	Years		*PavCNR12* genotype			
				1/1	1/2	1/3	2/3
N × E	Fruit wt (g)	2009	*N* ^a^	102	106	86	142
			Mean^b^	4.48^A^	4.41^A^	4.09^B^	3.69^C^
		2010	*N*	32	19	19	37
			Mean	4.76^A^	4.42^A,B^	4.28^B^	3.85^C^
		2011	*N*	44	40	43	52
			Mean	3.88^A^	3.65^B^	3.56^B^	3.41^B^
	Pit wt (g)	2011	*N*	44	40	43	52
			Mean	0.283^A^	0.255^B^	0.264^A,B^	0.272^A,B^
	Flesh wt (g)	2011	*N*	44	40	43	52
			Mean	3.60^A^	3.39^A,B^	3.30^B,C^	3.13^C^
	Mesocarp cell number^c^	2006	*N*	18	35	23	46
			Mean	38.0^A^	32.1^B^	31.9^B^	30.7^B^
		2007	*N*	19	41	26	48
			Mean	37.6^A^	32.7^B^	32.2^B^	31.0^B^

				1/1	1/2	2/2
R × L	Fruit wt (g)	2008	*N*	19	53	32
			Mean	9.34^A^	8.22^B^	6.52^C^
		2009	*N*	23	58	35
			Mean	8.67^A^	7.83^B^	6.25^C^
		2010	*N*	22	58	34
			Mean	8.29^A^	7.47^B^	6.28^C^

^a^Number of individuals

^b^Values marked with the same letter within a row are not significantly different (ANOVA, *P* > 0.05)

^c^Mesocarp cell number data is from Zhang et al. (2010)

Total fruit weight is the result of the combined weight of the pit and flesh. To determine whether the G2 fruit weight QTL is predominantly associated with flesh weight, the three *PavCNR12* alleles were evaluated for their association with flesh and pit weight (Table 1). Progeny individuals with the *PavCNR12*-*1/1* genotype showed the highest mean fruit weight and also the highest mean flesh weight. Progeny individuals with the *PavCNR12*-*2/3* genotype exhibited the lowest mean fruit weight and the lowest mean flesh weight. This suggested that the QTL effect on fruit size was mainly due to differences in the flesh rather than pit size.

The co-localization on G2 of a QTL for mesocarp cell number with the QTL for fruit size led to the presumption that differences in cell number might contribute to the differences in fruit size (Zhang et al. 2010). In the present study, mean mesocarp cell numbers were compared among the four *PavCNR12* progeny classes segregating in the N × E population (Table 1). Progeny with the *PavCNR12*-*1/1* genotype had a mean mesocarp cell number significantly higher than any other genotypic group in both years (38.0 and 37.6).

The R × L progeny population showed the mean fruit weight for the homozygous and heterozygous classes for the *PavCNR12* alleles 1 and 2 (Table 1). The mean values between the three genotypic classes were consistently significantly different (*P* < 0.05) from each other in all 3 years. Most notably, progeny with the homozygous genotype *PavCNR12*-*1/1* had the highest mean fruit weight, similar to that observed in N × E, while progeny with the homozygous genotype *PavCNR12*-*2/2* had the smallest mean fruit weight.

To test the likelihood of *PavCNR12* being the underlying gene responsible for the G2 fruit size QTL, additional G2 markers were developed between markers CPSCT038 and BPPCT034 and extending to the previously reported SSR MA007a (Olmstead et al. 2008). These marker scores, including *PavCNR12*, were analyzed along with fruit size data in a linear model ANOVA with additive effects and resulting probability values were scaled as –log_10_(*P*). The majority of markers in the region containing *PavCNR12* were significantly associated [*P* < 0.01 or −log_10_(*P*) > 2] with fruit size variation (Supplementary Fig. S3). In N × E, the most probable location of the fruit weight QTL standardized across 3 years was placed on marker G2SSR1576, immediately downstream of *PavCNR12.* This is also the most probable location of the flesh weight QTL. By comparison, the peak for mesocarp cell number was placed on RosCOS1634, which was located immediately upstream of *PavCNR12* (Supplementary Fig. S3). Interestingly, the analysis highlighted a second peak around marker BPPCT034 in the N × E population. While in most years this was a minor peak, in 2009 marker BPPCT034 showed the most significant association with fruit weight, suggesting a second fruit weight QTL in the N × E population. The strong association of fruit size variation over the region may be due to the small number of recombinant individuals for the region and the high effect of the underlying gene on fruit weight. The −log_10_(*P*) values for 2010 and 2011 were lower than those in 2009. This is because in 2010 and 2011 fewer individuals were evaluated, since only recombinant individuals and controls were studied for fruit size. The same analysis was carried out for the R × L progeny population (Supplementary Fig. S3). In this population, only one fruit weight QTL was found and the *PavCNR12* allele consistently corresponded to the most significant marker for mean fruit weight across all the 3 years of analysis (Table 1).

### Analysis of markers near *PcrCNR12* and *PcrCNR20* in sour cherry

For sour cherry, due to the difficulty in obtaining quality sequence data of amplicons derived from the *PcrCNR12* and *PcrCNR20* loci, two SSR markers were developed that are in close proximity to *PpCNR12* and *PpCNR20*. Based on the peach genome sequence, the first SSR developed was 18 kb downstream of *PpCNR12* (marker G2SSR1566) and the second SSR developed was 13 kb downstream of *PpCNR20* (G6SSR2208) (Supplementary Table S2). Due to the proximity to the *CNR* loci, these markers were used as proxies for the actual *CNR* alleles.

Fragment size differences for G2SSR1566 identified a total of three and seven SSR alleles in sweet and sour cherry, respectively (Supplementary Table S5). We inferred that the 250-bp fragment identified in both sweet and sour cherry was similar based on identical flanking SSR and SNP markers (Supplementary Table S6). This 250-bp fragment was associated with the sweet cherry *PavCNR12* allele 2, suggesting that this allele may also be present in sour cherry. SSR fragment sizes of 225 and 228 that were associated with alleles *PavCNR12*-*1* and *PavCNR12*-*3,* respectively, were not identified in sour cherry, suggesting that these alleles were not in the sour cherry germplasm evaluated. Fragment size differences for G6SSR2208 identified a total of four and five SSR alleles in sweet and sour cherry, respectively (Supplementary Table S5). Based on common SSR fragment sizes and surrounding SNP markers, two of the sour cherry SSR alleles (alleles 3 and 5) may be equivalent to those in sweet cherry (Supplementary Table S6).

### Association of the G2SSR1566 and G6SSR2208 alleles with fruit size in sour cherry

In sour cherry, mean fruit, pit and flesh weights were compared among individuals based upon the presence or absence of the seven G2SSR1566 alleles (Table 2). In the progeny, the presence or absence of allele 2 did not result in significant differences for any of the three phenotypic traits scored. However, significant phenotypic differences were associated with the alleles 4, 6, and most notably with allele 8. Allele 8 on its own had a highly significant effect on fruit, pit and flesh weight, where its absence was associated with an average increase in weight (Table 2). The largest mean differences for fruit and flesh weight were identified in those individuals with or without both alleles 7 and 8 (last column in Table 2). Progeny individuals that inherited both alleles displayed a mean fruit weight of 4.66 g and a mean flesh weight of 4.34 g, whereas those without the alleles had a mean fruit weight of 5.74 g and a mean flesh weight of 5.04 g. This represents an 18.8 and 19.6 % reduction in mean flesh weight and fruit weight for those individuals that have both alleles 7 and 8. Pit weight was not significantly associated with the simultaneous presence or absence of alleles 7 and 8.

**Table 2:** Phenotypic means for the presence or absence of the G2SSR1566 alleles (linked to *PcrCNR12*) and G6SSR2208 alleles (linked to *PcrCNR20*) summed over 274 sour cherry progeny individuals

	Fruit weight (g)			Pit weight (g)			Flesh weight (g)		
	*N* ^a^	Mean^b^	*P* value	*N* ^a^	Mean^b^	*P* value	*N* ^a^	Mean^b^	*P* value
*G2SSR1566 alleles*									
2/no 2	128/146	5.64^A^/5.30^A^	0.07	128/146	0.34^A^/0.34^A^	0.72	128/146	5.30^A^/4.96^A^	0.06
4/no 4	241/33	5.41^A^/5.85^B^	0.05	241/33	0.34^A^/0.36^A^	0.07	241/33	5.08^A^/5.49^A^	0.06
5/no 5	33/241	5.55^A^/5.55^A^	0.74	33/241	0.32^A^/0.34^A^	0.14	33/241	5.24^A^/5.10^A^	0.68
6/no 6	116/158	5.24^A^/5.62^B^	0.05	116/158	0.34^A^/0.34^A^	0.59	116/158	4.91^A^/5.28^B^	0.04
7/no 7	122/152	5.45^A^/5.47^A^	0.94	122/152	0.35^A^/0.33^A^	0.08	122/152	5.10^A^/5.14^A^	0.86
8/no 8	139/135	5.11^A^/5.81^B^	0.0002	139/135	0.33^A^/0.35^B^	0.008	139/135	4.78^A^/5.46^B^	0.0002
9/no 9	113/161	5.57^A^/5.38^A^	0.34	113/161	0.34^A^/0.34^A^	0.43	113/161	5.22^A^/5.05^A^	0.34
7 + 8/no 7 or 8	56/56	4.66^A^/5.74^B^	0.0004	56/56	0.33^A^/0.34^A^	0.43	56/56	4.34^A^/5.40^B^	0.0003
*G6SSR2208 alleles*									
1/no 1	85/171	5.23^A^/5.66^B^	0.05	85/171	0.32^A^/0.35^B^	0.03	85/171	4.91^A^/5.32^A^	0.06
2/no 2	40/215	5.09^A^/5.60^A^	0.08	40/215	0.33^A^/0.34^A^	0.25	40/215	4.76^A^/5.26^A^	0.08
3/no 3	194/63	5.69^A^/5.00^B^	0.0005	194/63	0.35^A^/0.31^B^	0.0004	194/63	5.34^A^/4.69^B^	0.0006
4/no 4	20/236	4.19^A^/5.64^B^	<0.0001	20/236	0.29^A^/0.35^B^	<0.0001	20/236	3.89^A^/5.29^B^	<0.0001
5/no 5	180/75	5.52^A^/5.55^A^	0.87	180/75	0.34^A^/0.35^A^	0.11	180/75	5.18^A^/5.20^A^	0.94

^a^Number of individuals

^b^Values marked with the same letter within a haplotype are not significantly different (ANOVA, *P* > 0.05)

For the G6SSR2208 alleles, the phenotypic means showed a significant increase for fruit, pit and flesh weight with the presence of allele 3 (Table 2). When considering that this allele might be shared between sweet and sour cherry, the effect on weight was consistent with the large-fruited G6 QTL allele in both species and its effect on pit weight. On the other hand, fruit, pit, and flesh weights were significantly higher when allele 4 was absent, suggesting a large negative effect on weight by this allele.

## D**iscussion**

### Cell number regulator genes and organ size

In the present study, we identified the *FW2.2/CNR* gene family characterized by the conserved PLAC8 domain in the genome of peach (Guo et al. 2010; Guo and Simmons 2011). Because of the role in fruit size in tomato, we sought to determine whether members of the *CNR* family might underlie fruit weight QTL in other species. The high colinearity within the *Prunus* genus permitted us to evaluate whether members of the *CNR* family in peach co-localize with important fruit weight QTL in sweet and sour cherry. We identified two *CNR* family members, *PavCNR12* and *PavCNR20*, as potential candidates to control fruit size in both sweet and sour cherry.

In plants, tomato *FW2.2* is the founding member of a family of genes controlling fruit size (Frary et al. 2000). *FW2.2* is shown to modulate cell proliferation in the carpel ovary; thus, its effect on fruit size is exerted by regulating cell number rather than cell size. Interestingly, the coding sequences of *FW2.2* alleles were identical, suggesting that the differences between the large- and small-fruited allele are based on the timing and level of gene expression rather than on changes in the protein structure or functionality. This hypothesis was supported by transgenic experiments in an artificial gene dosage series (Liu et al. 2003). *FW2.2* acts as a negative cell number regulator, as its dosage and level of expression are negatively correlated with the cell division activity in the early stages of fruit development.

The control of organ size and cell number by members of the *FW2.2/CNR* family could be a common regulatory mechanism in higher plants. Other members of the family Solanaceae possess overlapping fruit size QTL, suggesting conserved function of *FW2.2* in eggplant and pepper (Chaim et al. 2001; Doganlar et al. 2002). Additionally, a *FW2.2/CNR* family member in avocado may control fruit size by regulating cell proliferation (Dahan et al. 2010). Plant organ size in general is likely regulated by *CNR* genes. For example, the search for *FW2.2* members in maize led to the identification of a family of 13 *CNR* genes. Two of them, *ZmCNR1* and *ZmCNR2*, were shown to alter organ size (Guo et al. 2010). Over-expression of *ZmCNR1* resulted in a reduction of the overall plant stature, highlighting that it acts as a negative cell number regulator in multiple tissues; *ZmCNR2* expression level was negatively correlated with cell production, even though transgenic lines over-expressing *ZmCNR2* did not result in a phenotype (Guo et al. 2010). Another *FW2.2* family member regulates root nodule organogenesis in soybean (Libault et al. 2010). The expression of the soybean *FWL1* (*fw2.2*-*like 1*) is induced in root hair cells during nodulation and its silencing results in a reduction of nodule number, suggesting that *FWL1* acts as an initiator of organ development as a result of cell proliferation.

Some members of the *FW2.2/CNR* family are known to encode plasma membrane-bound proteins, showing common features in their tertiary structure which is made up of one or two trans-membrane helices surrounded by a cysteine- and proline-rich domain. They include proteins involved in the transport of metal cations through the plasma membrane, such as the cadmium transporters PCR and the calcium channels MCA. Similar to other known metal transporters, they act as homo-oligomers forming a complex able to bind and transport divalent cations (Song et al. 2010; Kurusu et al. 2012). The tobacco (*Nicotiana tabacum* L.) *MCA1* and *MCA2* genes encode putative Ca^2+^-permeable channels involved in the response to mechanical stress. Interestingly, over-expression of *NtMCA1* and *NtMCA2* in tobacco cells resulted in a significant reduction of cell proliferation (Kurusu et al. 2012), an effect that can be considered similar to that observed for *FW2.2*- and *ZmCNR1*-over-expressing lines of tomato and maize, respectively. Interestingly, tomato FW2.2 is found at the plasma membrane even though a role in cation transport has not yet been demonstrated (Cong and Tanksley 2006). Based on this finding, it can be hypothesized that cell proliferation is induced by *FW2.2/CNR* members via a modulation of the intracellular calcium concentration acting as a second messenger in signal transduction pathways controlling the cell cycle. However, further studies are needed to elucidate the mechanism by which *FW2.2/CNR* members exert their function.

### Amplification of the *CNR* genes in the plant kingdom

PLAC8 domain-containing proteins are known in animals and a variety of other eukaryotes. However, in plants the number of family members is larger than in other organisms. *Prunus* contains 23 *CNR* genes, which is more than in maize (12 members) (Guo et al. 2010) and tomato (19 members). While it is possible that not all *CNR* genes are correctly annotated, the high number in peach can be explained in part as a consequence of a series of recent tandem duplication events that produced a dense gene cluster on chromosome 1. Despite differences in plant gene copy numbers, the phylogenetic tree supports the hypothesis suggested by Guo et al. (2010) of a plant-specific expansion and radiation of *CNR* genes. Even though statistical support for a single plant-only cluster is low, sequences from animals and fungi are confined to the left-upper part of the tree (Fig. 2). Therefore, while the *FW2.2*/*CNR* gene family members might play a role within an ancient signal transduction pathway that evolved before the divergence of single- and multi-cellular organisms (Cong and Tanksley 2006), their duplication and diversification in plants may reflect the need to coordinate cell division activity within different tissues, organs and growth stages to a higher level of complexity than in animal and fungal systems.

### Evaluation of *CNR* genes as candidates for known cherry fruit size QTL

*FW2.2/CNR* gene family members are likely to underlie fruit size variation in other domesticated plants, such as those found in the Rosaceae family. Scorza et al. (1991) compared large- and small-fruited peach varieties in terms of cell number and cell size; while cell sizes were similar among all cultivars, the large-fruited genotypes exhibited a higher number of cells at all developmental stages, suggesting that the main mechanism by which fruit size is determined is cell proliferation in the early stages of ovary development. Similarly, Olmstead et al. (2007) reported that differences in cherry fruit size associated with domestication and modern breeding are mainly due to increases in cell number rather than cell size. These findings support the hypothesis that *FW2.2/CNR* genes could be involved in the control of fruit size in *Prunus*. A peach *CNR* homolog, *PpCNR12*, localized in the cherry G2 QTL interval and the position was confirmed by mapping *PavCNR12* in the N × E and R × L populations. The position of *PavCNR12* was consistent with the high fruit size QTL LOD scores, even though the most significant markers for mesocarp cell number and fruit weight in some years were found for those that were located immediately upstream and downstream of *PavCNR12*. On the other hand, *PavCNR12* is clearly the most significant marker associated with fruit size in the R × L population. This is despite the fact that the population is much smaller, yet the parents are more closely related to one another than in the N × E population. Fruit size of sour cherry is also likely controlled by *PcrCNR12* since a closely linked marker shows association with fruit size.

Because of the wider cross and likelihood of many minor QTL, and despite the large population size of 557 individuals in the N × E population, the number of recombinants within this short region was too low to conduct a high-resolution fine-mapping analysis of the QTL to a single gene. Nevertheless, further support for the role of *PavCNR12* in controlling fruit size in both sweet cherry populations was demonstrated by the haplotypes that were found. Specifically, *PavCNR12*-*1* was associated with the large-fruited QTL allele in both progenies and, conversely, *PavCNR12*-*2* was consistently associated with a small-fruited allele. The third allele, *PavCNR12*-*3*, was associated with the least favorable QTL allele in the N × E progeny. It is thus possible that the *PavCNR12* alleles differentially contribute to fruit size. Similar to the tomato *FW2.2*, no differences in the protein-coding region were found among the three alleles. Thus, the proposed effect of the *PavCNR12* alleles might depend on the regulation of expression. Consistent with this hypothesis, the highest variation in the sequences was found in the promoter regions.

The analysis of the G6 candidate gene, *PavCNR20,* supported the presence of a divergent allele in New York 54, differentiating this genotype from all the other tested cultivars. Accordingly, only New York 54 was found to bear the unfavorable QTL allele, supporting the notion that the favorable allele is fixed in cultivated varieties during the domestication process of this species. Analysis of a SSR marker in the same region suggested the presence of the same QTL in sour cherry as well. Further analyses are needed to ascertain whether *PavCNR20* could actually be responsible for the QTL effect in both species. Nevertheless, if the favorable allele(s) is fixed in sweet cherry domesticated varieties, the practical importance of this QTL will be limited to populations derived from crosses with wild genotypes. In other words, *PavCNR20* could be considered a gene associated with the domestication process.

### Enabling marker-assisted breeding

The QTL on G2 is the most important QTL involved in the control of fruit size in modern cherry germplasm, explaining the highest portion of the phenotypic variation (Zhang et al. 2010; Dirlewanger et al. 2009). While definitive proof of whether *PavCNR12* controls fruit size awaits further experimentation, the co-localization of *PavCNR12* with the G2 QTL peak and the association of its haplotypes with the QTL effects support the hypothesis that both *PavCNR12* and *PcrCNR12* control fruit size in sweet and sour cherry, respectively. Allelic variation at the cherry *CNR12* locus can be used to select from sweet cherry R × L and N × E populations those individuals that are homozygous for the *PavCNR12*-*1* allele, which showed a mean fruit weight 16 and 9 % higher than the mean value for their respective entire populations. For sour cherry, selection against alleles 4, 6 and in particular allele 8, and for allele 2 of marker G2SSR1566, should result in progeny exhibiting larger fruit size and flesh weight.

The fruit size allele on G6 is less important for sweet cherry breeding programs, as the favorable allele is fixed in the cultivated germplasm. On the other hand, this could be an important marker for sour cherry breeding, as several putative alleles were identified with a significant effect on fruit size, possibly originating from the undomesticated progenitor species, *P. fruticosa*. In particular, selection for allele 3 and against allele 4 of marker G6SSR2208 should yield progeny with larger fruit size and flesh weight.

In summary, genetic and sequence data suggested that two of the peach *CNR* gene family members are excellent candidate genes for two fruit size QTL in sweet and sour cherry. The finding that the increase in fleshy ovary size in both tomato and cherry associated with domestication may be due to changes in members of the same ancestral gene family supports the notion that similar phenotypic changes exhibited by independently domesticated taxa may have a common genetic basis.

## Electronic supplementary material

Below is the link to the electronic supplementary material.

Supplementary material 1 (DOCX 33 kb)

Supplementary material 2 Structure of the 23 peach *CNR* genes; exons and introns are represented as thick and thin lines, respectively (TIFF 161 kb)

Supplementary material 3 Haplotypes identified for the *CNR12* region on G2 in sweet cherry with the peach physical map locations of the markers/polymorphisms identified. SNPs differentiating *PavCNR* alleles 1, 2, and 3 are highlighted in yellow, blue and green, respectively (XLSX 22 kb)

Supplementary material 4 Correlation between markers spanning the sweet cherry LG2 QTL region and fruit size, calculated by ANOVA; data for fruit weight (FW) and mesocarp cell number (MCN) from different years are reported as dashed lines; solid lines represent data standardized across different years of analysis (std). Flesh weight data is reported for the only year in which it was determined (2011) (TIFF 581 kb)
