# Supplementary material for: Cell number regulator genes in Prunus provide candidate genes for the control of fruit size in sweet and sour cherry
Source: Mol Breed. 2013 Apr 30;32(2):311–26. doi: 10.1007/s11032-013-9872-6 (PMC3748327; doi:10.1007/s11032-013-9872-6)
Supplement: Supplementary file 1 — Supplementary material 1 (DOCX 33 kb) [file 11032_2013_9872_MOESM1_ESM.docx]

Molecular Breeding

**Cell Number Regulator genes in *Prunus* provide candidate genes for the control of fruit size in sweet and sour cherry**

De Franceschi P., Stegmeir T., Cabrera A., van der Knaap E., Rosyara U.R., Sebolt A.M., Dondini L., Dirlewanger E., Quero-Garcia J., Campoy J.A., Iezzoni A.F.*

* Corresponding author

Michigan State University, East Lansing, Michigan, USA

iezzoni@msu.edu

**Supplementary Table S1.** Primers used for sequencing *PavCNR12* and *PavCNR20*; M13 tails for sequencing are indicated in *italics*.

| Primer | Tail | Sequence 5’ – 3’ |
| --- | --- | --- |
| CNR12-C1 for | M13F | *cacgacgttgtaaaacgac*agtggcaacataactggatg |
| CNR12-C1 rev | M13R | *caggaaacagctatgacc*aagtttggtgttgaacttgtct |
| CNR12-C2 for | M13F | *cacgacgttgtaaaacgac*ttgccataaatagatatccaaaa |
| CNR12-C2 rev | M13R | *caggaaacagctatgacc*gatttccatcagccatctg |
| CNR12-C3 for | M13F | *cacgacgttgtaaaacgac*cagatggctgatggaaatc |
| CNR12-C3 rev | M13R | *caggaaacagctatgacc*cttccaaatcattaggtcaca |
| CNR12-C4 for | M13F | *cacgacgttgtaaaacgac*tggcccacttatgatgatt |
| CNR12-C4 rev | M13R | *caggaaacagctatgacc*aaagtagggcctatcaccaa |
| CNR12-C5 for | M13F | *cacgacgttgtaaaacgac*caaatggcttttggtgatag |
| CNR12-C5 rev | M13R | *caggaaacagctatgacc*agcagcatcattgtgttcat |
| CNR12-C6 for | M13F | *cacgacgttgtaaaacgac*tcatgaacacaatgatgctg |
| CNR12-C6 rev | M13R | *caggaaacagctatgacc*ttgaagtaggagtttcttagtttga |
| CNR20-C1 for | M13F | *cacgacgttgtaaaacgac*gccttattaatttgctcaacc |
| CNR20-C1 rev | M13R | *caggaaacagctatgacc*aaaacatctactgccgcttt |
| CNR20-C2 for | M13F | *cacgacgttgtaaaacgac*cgctagtaaagggcaaataa |
| CNR20-C2 rev | M13R | *caggaaacagctatgacc*aaaatcatccagaaccctgt |
| CNR20-C3 for | M13F | *cacgacgttgtaaaacgac*accattctgcaatttggtat |
| CNR20-C3 rev | M13R | *caggaaacagctatgacc*caaacgtcccaggagtagat |
| CNR20-C4 for | M13F | *cacgacgttgtaaaacgac*gtgatctggttaggccttct |
| CNR20-C4 rev | M13R | *caggaaacagctatgacc*agaattggttgagcattgaa |
| CNR20-C5 for | M13F | *cacgacgttgtaaaacgac*agctttgtggaagatgaggt |
| CNR20-C5 rev | M13R | *caggaaacagctatgacc*atcttgaaccatttgccatt |
| CNR20-C6 for | M13F | *cacgacgttgtaaaacgac*atgtcattttaaacgaacttagtaat |
| CNR20-C6 rev | M13R | *caggaaacagctatgacc*ggttgcataacttctcgatt |
| M13 for | - | cacgacgttgtaaaacgac |
| M13 rev | - | caggaaacagctatgacc |

**Supplementary Table S2.** SSR and SNP markers developed in this study.

| Marker | Primer Sequence 5’ – 3’ | Ta | Amplification sizes | Species Origin | Peach physical map location (Mb) |
| --- | --- | --- | --- | --- | --- |
| G2SSR1576_F | AAAGGAGTTGCGACATTCAA | 60° | ~270-290bp | *P. persica* | Scaffold 2 (15.761) |
| G2SSR1576_R | TCCATTGCACAAGAGTTTGC |  |  |  |  |
| G2SSR1580_F | CGTTTTCCCATGTCAGCTCT | 60° | ~200-210bp | *P. persica* | Scaffold 2 (15.809) |
| G2SSR1580_R | TTCGCTCTTCCACTCCATCT |  |  |  |  |
| G2SSR1610_F | GAAAACCCTAAAACCCTAACC | 56° | ~240-250bp | *P. persica* | Scaffold 2 (16.105) |
| G2SSR1610_R | GGTGGGTAAAACTGTCATATTG |  |  |  |  |
| G2SSR1672_F | CTTGCCGTGAGAGAATCCTT | 60° | ~208-241bp | *P. persica* | Scaffold 2 (16.723) |
| G2SSR1672_R | GCTGGAGAAAGAATTAGGAAAATG |  |  |  |  |
| G2SSR1678_F | TAGACTGTGAAGGAACAAGACC | 56° | ~242-274bp | *P. persica* | Scaffold 2 (16.782) |
| G2SSR1678_R | TCTCTTCTCCAGGTTTACACAC |  |  |  |  |
| G2SSR1675_F | TTAAATTCCTCCAGACCTAACC | 56° | ~206-212bp | *P. persica* | Scaffold 2 (16.754) |
| G2SSR1675_R | GGTGGTAGTGGTGTCAGTTTTA |  |  |  |  |
| G2SSR1818_F | ATGCTGTTCGGAGGTCTGTT | 60° | ~275-280bp | *P. persica* | Scaffold 2 (18.180) |
| G2SSR1818_R | TGAATGCATGTGTGTACGTG |  |  |  |  |
| G2SSR1823_F | GAGGCAAAGCATGATCCAGT | 60° | ~234-236bp | *P. persica* | Scaffold 2 (18.230) |
| G2SSR1823_R | CTCATGTTCACCAGCCTTCA |  |  |  |  |
| G2SSR1864_F | TTTCGATCCCATTGTTGAGG | 60° | ~260-270bp | *P. persica* | Scaffold 2 (18.644) |
| G2SSR1864_R | ACACCGGGCAACTGTACTTC |  |  |  |  |
| G2SSR1623_F^1^ | ATGGCTTGCTTTCCCACTTA | 56° | ~470bp | *P. persica* | Scaffold 2 (16.236) |
| G2SSR1623_R^1^ | GCAGAGGACAACATTAAAAATGC |  |  |  |  |
| G2SNP1623_F^1^ | TCTAATCTTGGCATCAAGC | 56° | C/T | *P. persica* | Scaffold 2 (16.236) |
| G2SNP1623_R^1^ | TCTAATCTTGGCATCAAGT |  |  |  |  |
| G2SSR1566_F | ATTCCTCTTTCGCCTTCC | 54° | ~210-250bp | *P. persica* | Scaffold 2 (15.667) |
| G2SSR1566_R | GGAGCTATTCGAGTCGTCA |  |  |  |  |
| G6SSR2208_F | CAACGGCCTATACGTAGTTGAG | 58° | ~120-130bp | *P. persica* | Scaffold 6 (22.083) |
| G6SSR2208_R | CTTACCAACATTGGGAGACAAA |  |  |  |  |

^1^ SNP marker, for allele-specific primer extension (ASPE) of the SNP 334 on G2SSR1623_F and R PCR product was genotyped by the APSE with primers G2SNP1623_F and R using the Luminex 200 genotyping platform.

**Supplementary Table S3.** *CNR* gene family members in the peach genome (also see Fig. 1)

| Gene | Transcript Name | Position | | |
| --- | --- | --- | --- | --- |
|  |  | Scaffold | Transcript Start (bp) | Transcript End (bp) |
| PpCNR01 | ppa016412m | 1 | 3056759 | 3057893 |
| PpCNR02 | ppa019128m | 1 | 3067215 | 3068180 |
| PpCNR03 | ppa017629m | 1 | 3074596 | 3075380 |
| PpCNR04 | ppa015286m | 1 | 3085350 | 3086043 |
| PpCNR05 | ppa017874m | 1 | 3098959 | 3099875 |
| PpCNR06 | ppa020240m | 1 | 3109834 | 3110729 |
| PpCNR07 | ppa020310m | 1 | 3117341 | 3118324 |
| PpCNR08 | ppa017482m | 1 | 3137493 | 3138669 |
| PpCNR09 | ppa018965m | 1 | 29037152 | 29038193 |
| PpCNR10 | ppa018556m | 1 | 29038884 | 29039662 |
| PpCNR11 | ppa011430m | 1 | 30408757 | 30410218 |
| PpCNR12 | ppa026136m | 2 | 15647989 | 15650767 |
| PpCNR13 | ppa019431m | 2 | 23206643 | 23208417 |
| PpCNR14 | ppa005753m | 2 | 23383585 | 23384937 |
| PpCNR15 | ppa010809m | 2 | 24205436 | 24207526 |
| PpCNR16 | ppa023277m | 3 | 3774129 | 3774661 |
| PpCNR17 | ppa010659m | 4 | 1574883 | 1576896 |
| PpCNR18 | ppa017696m | 5 | 17128673 | 17129593 |
| PpCNR19 | ppa021734m | 5 | 17130224 | 17132195 |
| PpCNR20 | ppa008853m | 6 | 22070836 | 22074112 |
| PpCNR21 | ppa011977m | 7 | 877717 | 880389 |
| PpCNR22 | ppa006351m | 7 | 17754476 | 17758740 |
| PpCNR23 | ppa023567m | 8 | 14205694 | 14207167 |

**Supplementary Table S4.** *PavCNR12* genotypes for a diverse set of 17 sweet cherry cultivars (Cabrera et al. 2012). The SNPs that differentiate the three *PavCNR12* alleles are presented in Supplementary Figure S1.

| Cultivar | *PavCNR12* genotype |
| --- | --- |
| Ambrunes | 1/1 |
| Bing | 1/2 |
| Chelan | 1/3 |
| Cowiche | 1/1 |
| Cristobalina | 1/3^1^ |
| Emperor Francis | 1/2 |
| Krupnoplodnaya | 1/1 |
| Lambert | 1/3 |
| Lapins | 1/2 |
| Napoleon | 1/2 |
| New York 54 | 1/3 |
| Regina | 1/2 |
| Sam | 1/1 |
| Schmidt | 1/1 |
| Tieton | 1/1 |
| Van | 1/2 |
| Windsor | 1/1 |

^1^ The *PavCNR12-3* in ‘Cristobalina’ has an additional SNP within the third intron compared to the other three *PavCNR12* alleles.

**Supplementary Table S5.** Fragment sizes for SSR markers used to discriminate the genomic regions containing *PcrCNR12* and *PcrCNR20* in sweet and sour cherry.

| Germplasm | Linkage Group | SSR Allele | Marker | Fragment size (bp) |
| --- | --- | --- | --- | --- |
| Sweet | 2 | 1 | G2SSR1566 | 225 |
|  | 2 | 2^1^ | G2SSR1566 | 250 |
|  | 2 | 3 | G2SSR1566 | 228 |
|  | 6 | 3^2^ | G6SSR2208 | 124 |
|  | 6 | 5^3^ | G6SSR2208 | 130 |
|  | 6 | 6 | G6SSR2208 | 116/118/122^4^ |
|  | 6 | 7 | G6SSR2208 | 128 |
| Sour | 2 | 2^1^ | G2SSR1566 | 250 |
|  | 2 | 4 | G2SSR1566 | 210 |
|  | 2 | 5 | G2SSR1566 | 212 |
|  | 2 | 6 | G2SSR1566 | 235 |
|  | 2 | 7 | G2SSR1566 | 239 |
|  | 2 | 8 | G2SSR1566 | 225 |
|  | 2 | 9 | G2SSR1566 | 228 |
|  | 6 | 1 | G6SSR2208 | 120 |
|  | 6 | 2 | G6SSR2208 | 122 |
|  | 6 | 3^2^ | G6SSR2208 | 124 |
|  | 6 | 4 | G6SSR2208 | 126 |
|  | 6 | 5^3^ | G6SSR2208 | 130 |
| ^1, 2, 3^ SSR fragments from sweet and sour cherry that had equivalent bp sizes were given the same allele number if flanking marker data was identical. For G2SSR1566, 11 flanking markers were examined, the SSRs CPSCT038 and BPPCT034 and nine SNP markers (Supplementary Table S6). For G6SSR2208, 38 flanking SNP markers were considered (Supplementary Table S6). | | | | |
| ^4^ These three fragments are inherited together. | | |  |  |

**Supplementary Table S6.**  Peach physical map locations for *PpCNR12*, *PpCNR20* and their associated SSRs (G2SSR1566 and G6SSR2208, respectively) along with SNPs used to define the *CNR* allelic variants between sweet and sour cherry.

| Scaffold | Marker | Peach Physical Map Location (bp)^1^ |
| --- | --- | --- |
| 2 | CPSCT038 | 2_15057199 |
|  | ss490549172^2^ | 2_15084429 |
|  | ss490549216 | 2_15337787 |
|  | ss490549227 | 2_15372418 |
|  | ss490549238 | 2_15492297 |
|  | *PpCNR12* | 2_15647989 |
|  | G2SSR1566 | 2_15666975 |
|  | ss490549287 | 2_15747822 |
|  | ss490549311 | 2_15846482 |
|  | ss490549331 | 2_15894385 |
|  | ss490549379 | 2_16118423 |
|  | ss490549383 | 2_16142700 |
|  | BPPCT034 | 2_16491338 |
| 6 | *PpCNR20* | 6_22070836 |
|  | G6SSR2208 | 6_22083802 |
|  | ss490550132 | 6_22122175 |
|  | ss490556003 | 6_22194565 |
|  | ss490550135 | 6_22481221 |
|  | ss490556011 | 6_22483330 |
|  | ss490556014 | 6_22578566 |
|  | ss490556018 | 6_22682473 |
|  | ss490550143 | 6_22731299 |
|  | ss490558923 | 6_22953307 |
|  | ss490556027 | 6_22969579 |
|  | ss490556030 | 6_23001313 |
|  | ss490550163 | 6_23103755 |
|  | ss490550167 | 6_23117929 |
|  | ss490556045 | 6_23121232 |
|  | ss490550171 | 6_23125937 |
|  | ss490556048 | 6_23138881 |
|  | ss490550179 | 6_23368978 |
|  | ss490556080 | 6_23460054 |
|  | ss490556083 | 6_23466387 |
|  | ss490550183 | 6_23491156 |
|  | ss490550187 | 6_23515096 |
|  | ss490556092 | 6_23550234 |
|  | ss490550192 | 6_23617261 |
|  | ss490550196 | 6_23656729 |
|  | ss490550200 | 6_23717104 |
|  | ss490559356 | 6_23740186 |
|  | ss490556117 | 6_23776068 |
|  | ss490550208 | 6_23799947 |
|  | ss490559289 | 6_23810925 |
|  | ss490556126 | 6_23851662 |
|  | ss490556129 | 6_23925194 |
|  | ss490550216 | 6_24251557 |
|  | ss490556147 | 6_24311905 |
|  | ss490559115 | 6_24324785 |
|  | ss490550220 | 6_24433974 |
|  | ss490556163 | 6_24593485 |
|  | ss490556173 | 6_24757894 |
|  | ss490550235 | 6_24770664 |
|  | ss490556176 | 6_24822456 |
| ^1^Physical map locations are based on the peach genome sequence v1.0 (International Peach Genome Initiative; <http://www.rosaceae.org/peach/genome>)  ^2^ SNP are described in Peace et al. (2012) | | |
