# Supplementary figures and images for: Cell number regulator genes in Prunus provide candidate genes for the control of fruit size in sweet and sour cherry
Source: Mol Breed. 2013 Apr 30;32(2):311–26. doi: 10.1007/s11032-013-9872-6 (PMC3748327; doi:10.1007/s11032-013-9872-6)

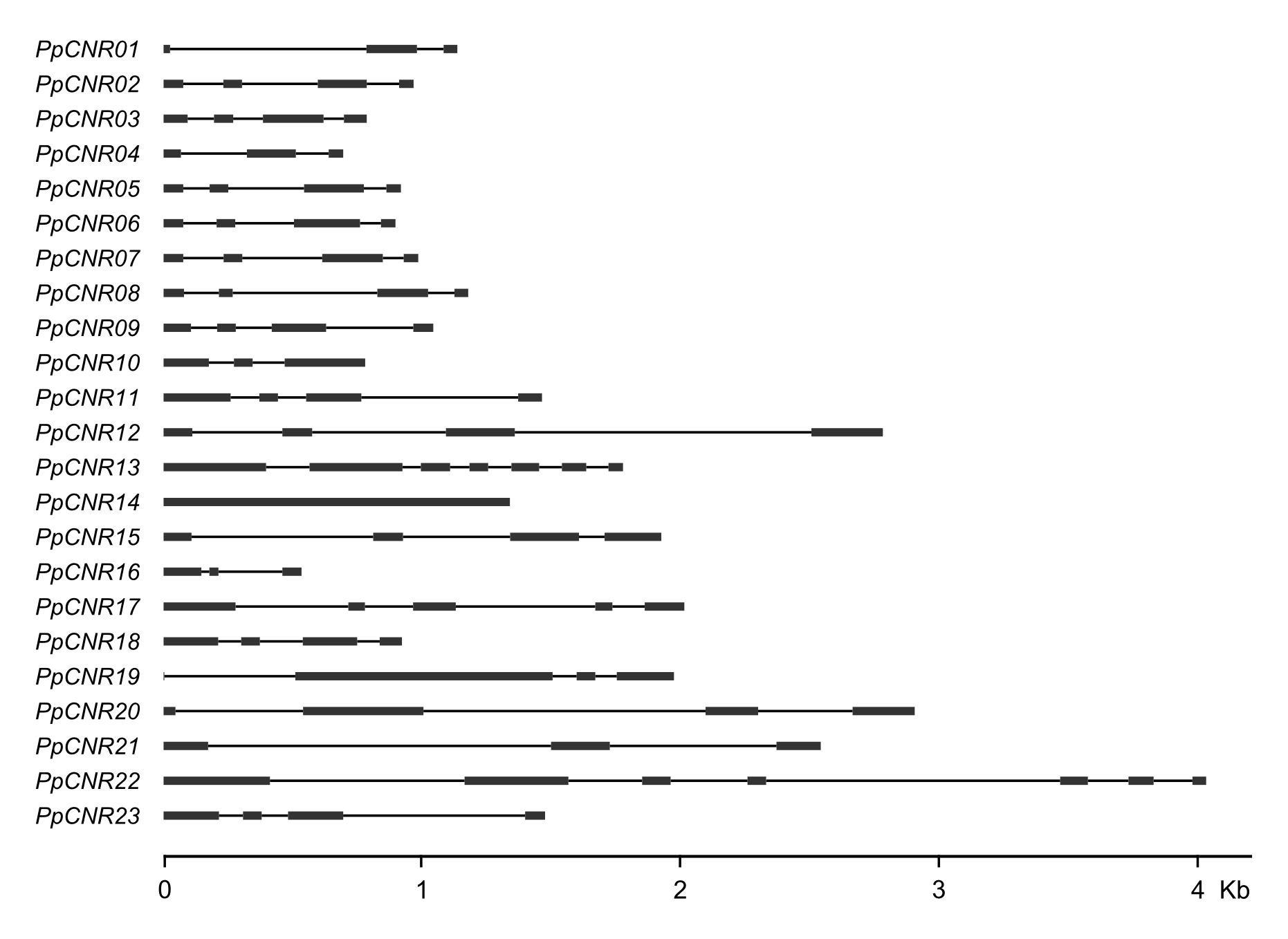

Supplement: Supplementary file 2 — Supplementary material 2 Structure of the 23 peach CNR genes; exons and introns are represented as thick and thin lines, respectively (TIFF 161 kb) [file 11032_2013_9872_MOESM2_ESM.tif]

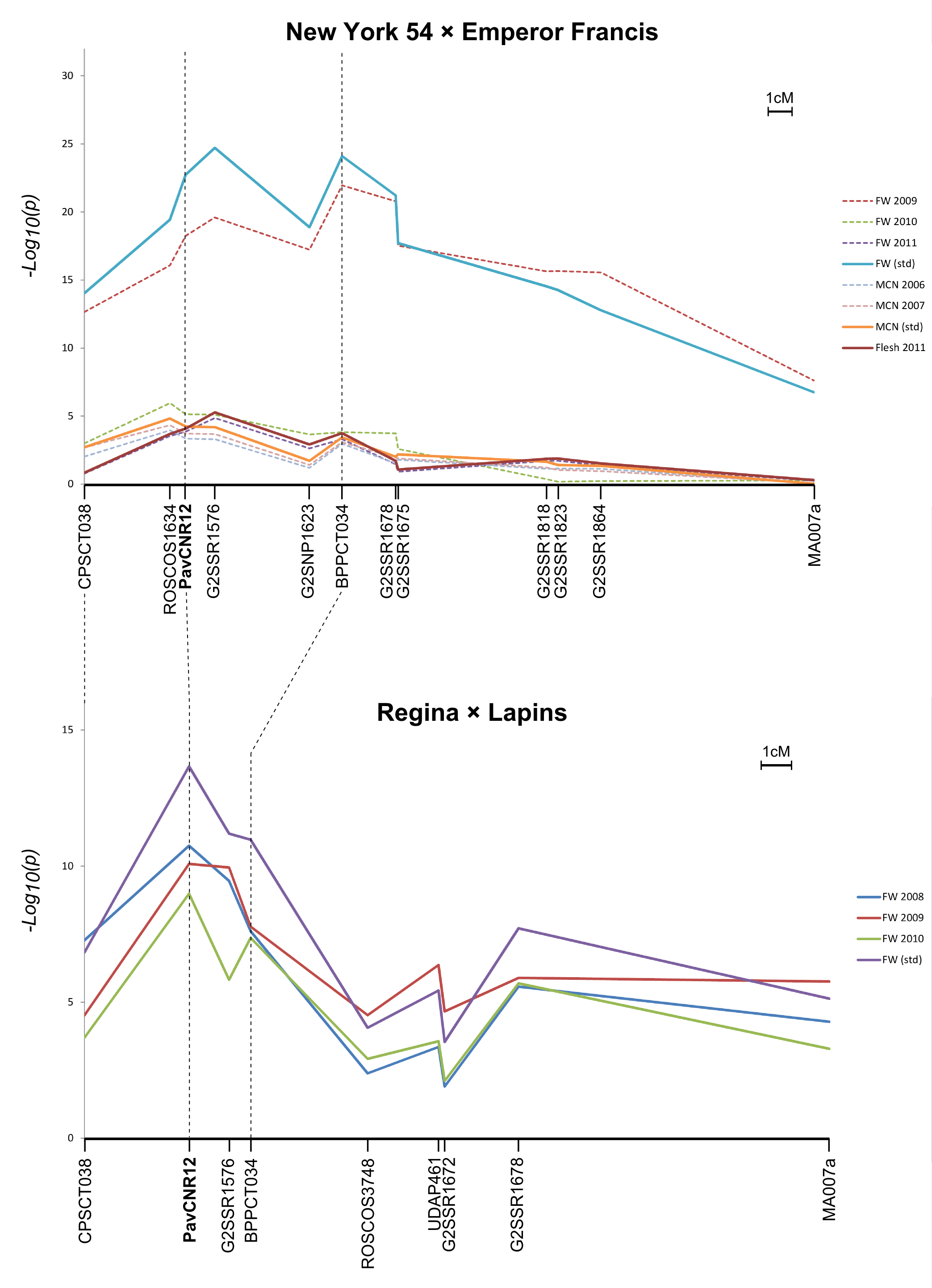

Supplement: Supplementary file 4 — Supplementary material 4 Correlation between markers spanning the sweet cherry LG2 QTL region and fruit size, calculated by ANOVA; data for fruit weight (FW) and mesocarp cell number (MCN) from different years are reported as dashed lines; solid lines represent data standardized across different years of analysis (std). Flesh weight data is reported for the only year in which it was determined (2011) (TIFF 581 kb) [file 11032_2013_9872_MOESM4_ESM.tif]
